# Tracking DNA-based antigen-specific T cell receptors during progression to type 1 diabetes

**DOI:** 10.1126/sciadv.adj6975

**Published:** 2023-12-08

**Authors:** Angela M. Mitchell, Erin E. Baschal, Kristen A. McDaniel, Theodore Fleury, Hyelin Choi, Laura Pyle, Liping Yu, Marian J. Rewers, Maki Nakayama, Aaron W. Michels

**Affiliations:** ^1^ Barbara Davis Center for Diabetes, University of Colorado School of Medicine, Aurora, CO, USA.; ^2^ Department of Biostatistics and Informatics, University of Colorado School of Public Health, Aurora, CO, USA.; ^3^ Department of Pediatrics, University of Colorado School of Medicine, Aurora, CO, USA.; ^4^ Department of Medicine, University of Colorado School of Medicine, Aurora, CO, USA.; ^5^ Department of Immunology, University of Colorado School of Medicine, Aurora, CO, USA.

## Abstract

T cells targeting self-proteins are important mediators in autoimmune diseases. T cells express unique cell-surface receptors (TCRs) that recognize peptides presented by major histocompatibility molecules. TCRs have been identified from blood and pancreatic islets of individuals with type 1 diabetes (T1D). Here, we tracked ~1700 known antigen-specific TCR sequences, islet antigen or viral reactive, in bulk TCRβ sequencing from longitudinal blood DNA samples in at-risk cases who progressed to T1D, age/sex/human leukocyte antigen–matched controls, and a new-onset T1D cohort. Shared and frequent antigen-specific TCRβ sequences were identified in all three cohorts, and viral sequences were present across all ages. Islet sequences had different patterns of accumulation based upon antigen specificity in the at-risk cases. Furthermore, 73 islet-antigen TCRβ sequences were present in higher frequencies and numbers in T1D samples relative to controls. The total number of these disease-associated TCRβ sequences inversely correlated with age at clinical diagnosis, indicating the potential to use disease-relevant TCR sequences as biomarkers in autoimmune disorders.

## Teaser

Type 1 diabetes relevant T cell receptors in blood can be tracked through disease development and correlate to age at diagnosis.

## INTRODUCTION

T cells and their unique cell surface receptors play a crucial role in adaptive immune responses by recognizing peptides presented by major histocompatibility complex molecules. T cell receptors (TCRs) are highly diverse as a result of recombination of variable (V), diversity (D), and joining (J) segments for TCRβ chains and V/J segments for TCRα chains, thus allowing for recognition of millions of unique antigens. The diversity of the α/βTCR repertoire is estimated at over 10^8^ unique α/βTCRs in a given individual using next generation–based sequencing techniques (*1*), which originate from a larger pool of possible rearrangements (*2*, *3*). By sequencing TCRβ chains from peripheral blood DNA, highly accurate assessments on prior pathogen exposure to cytomegalovirus (CMV) and severe acute respiratory syndrome coronavirus 2 (SARS-CoV-2) can be determined (*4*, *5*). However, similar comprehensive evaluations for self-reactive TCR sequences in autoimmunity has yet to be evaluated.

Type 1 diabetes (T1D) is an autoimmune disease that results from the immune-mediated destruction of insulin-producing β cells within pancreatic islets that develops in distinct stages before the onset of clinical symptoms (*6*). The preclinical stages of T1D generally occur over a period of years and are characterized by the presence of islet autoantibodies in peripheral blood that target insulin and pancreatic β cell proteins, the subsequent development of impaired glucose tolerance, and then clinical T1D onset marked by hyperglycemia and the need for lifelong insulin treatment (*7*). As both CD4 and CD8 T cells play a critical role in disease pathogenesis, many approaches have been used to identify and detect islet antigen–specific T cells, including single-cell cloning, fluorescent tetramer/multimer staining, proliferation assays, cytokine enzyme-linked immunospot assays, and producing TCR transductants or avatars expressing a single receptor (*8*). These approaches led to the discovery of numerous self-reactive T cells that respond to different β cell antigens (*9*). However, monitoring islet antigen–specific T cells has proven challenging, especially in young children, due to large blood volumes required to detect infrequent autoreactive T cells and the need for fresh samples to have optimal results with many assays (*10*). Monitoring these islet antigen–specific TCR sequences during the preclinical stages of T1D may provide important insights into disease activity before the manifestation of clinical symptoms.

Previously, we deep-sequenced the TCRβ chain repertoires from longitudinal peripheral blood DNA samples in children starting very early in life who were genetically at risk for developing T1D as part of the Diabetes Autoimmunity Study in the Young (DAISY) (*11*). From this immunoreceptor sequencing, we identified TCRs specific for influenza and those reactive to insulin and its precursor, preproinsulin (PPI). The PPI-reactive sequences came from CD4 and CD8 T cells within the islet infiltrate of multiple T1D organ donors with 15 of 44 (34%) TCRβ sequences (identical V, J, and CDR3) shared among at-risk T1D individuals. These PPI sequences were more common and frequent in number in those who progressed to T1D compared to age/sex/human leukocyte antigen (HLA)–matched controls who did not develop islet autoantibodies or diabetes. In addition, TCRβ sequencing in a separate cohort of patients with new-onset T1D validated the presence of these PPI sequences. This previous work, evaluating only 44 PPI-specific TCRs, implicated the potential of TCR usage as a surrogate to identify self-antigen–specific T cells. Here, we expand to thousands of other islet antigen–specific TCR sequences to evaluate the hypothesis that there are shared and frequent islet antigen–specific TCRβ sequences that are unique to individuals progressing through the stages of T1D development.

In this study, we identified islet and viral antigen–specific CD4 and CD8 TCR sequences from the published literature, which resulted in over 1700 TCRβ sequences of interest. We then searched for the presence of these antigen-specific TCRβ sequences in our bulk deep-sequencing results from peripheral blood DNA samples in cases that progressed to clinical T1D, age/sex/HLA-matched controls, and a separate new-onset T1D cohort (more than 50 million TCRβ sequences in total). We were able to identify shared and frequent sequences from this list, with islet antigen–specific TCRβ sequences showing differential patterns of accumulation as T1D progressed on the basis of whether they were derived from a CD8 or CD4 T cell and the islet protein targeted. CD4 PPI-specific TCRβs accumulated in number as T1D progressed, while these sequences decreased with age in controls. Islet peptide–specific CD8 TCRβs were present throughout T1D development and contracted in controls over time. In addition, we were able to select 73 islet antigen–reactive TCRβ sequences that were more commonly shared and with higher template numbers in the at-risk cases and new-onset T1D individuals compared to controls; these TCRβs were deemed T1D disease–associated. Expressing a higher total number of disease-associated TCRβ sequences correlated with an earlier age at clinical T1D onset, signifying the relevance of tracking disease-associated self-reactive TCR sequences in peripheral blood to monitor disease progression in T1D and potentially other autoimmune disorders.

## RESULTS

### Antigen-specific TCR sequences can be identified within bulk TCRβ chain repertoires from individuals genetically at risk and with new-onset T1D

**Fig. 1.**
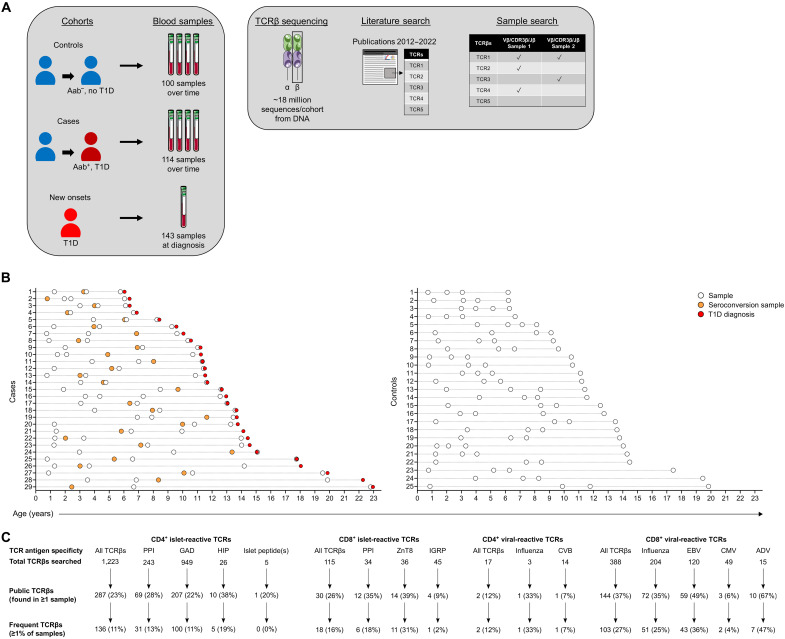
Antigen-specific TCR sequences within bulk TCRβ chain repertoires from individuals genetically at risk and with new-onset T1D. (**A**) Study design showing the three patient cohorts and number of peripheral blood samples from each cohort used to perform bulk TCRβ chain sequencing (~50 million total sequences). This large database of TCRβ sequences was queried using a curated list of 1743 antigen-specific TCR sequences, islet antigen or viral protein reactive, that was compiled from the published literature. (**B**) Graphs displaying the samples obtained by age from cases during progression to clinical T1D (left) and age-matched controls (right). Each row is a participant, with open circles indicating a sample. In preclinical T1D cases, orange circles indicate the sample at which the participant became islet autoantibody positive (seroconversion), and red circles indicate the age of clinical T1D onset. (**C**) Shown are the results of the sample search using the curated list of known antigen-specific TCRβ sequences, grouped by T cell subset and antigen specificity. Public TCRβ sequences were found in at least one sample, and frequent TCRβ sequences were found in at least 1% of the samples (i.e., >4 samples of 357) from any cohort.

To assess the bulk TCR repertoires during childhood, we previously performed deep sequencing of TCRβ chains using peripheral blood DNA samples from participants in a prospective birth cohort study following genetically at-risk children for the development of T1D, along with an additional cohort of patients with new-onset T1D (*11*). This database of TCRβ sequences contains more than 50 million sequences from (i) individuals with genetic risk for T1D conferred through HLA-DR-DQ alleles that developed islet autoantibodies [e.g., those directed to insulin, glutamic acid decarboxylase (GAD), tyrosine phosphatase–related islet antigen-2, and zinc transporter 8 (ZnT8)] and subsequently clinical T1D (*n* = 29 participants with 114 samples, termed cases), (ii) age/sex/HLA-matched children who did not develop islet autoantibodies or T1D (*n* = 25 participants with 100 samples, termed controls), and (iii) a separate cohort of patients with new-onset T1D sampled once at clinical diagnosis (*n* = 143 samples; Fig. 1A). Notably, cases were sequenced four times during their progression to T1D, and controls were age-matched to cases and sequenced four times at each peripheral blood sample (Fig. 1B). Overall, all three cohorts were matched for sex, ethnicity, and HLA-DR-DQ genotypes that confer T1D genetic risk, with no statistical differences between the groups, and new-onset T1D samples were well matched for age to the final samples from both cases and controls (Table 1). Similar numbers of TCRβ sequences, termed templates for an individual sequence, were obtained from the samples in each cohort (Table 1 and fig. S1). In addition, the TCRβ repertoires become more clonal over time in both cases and controls with no difference in Simpson productive clonality metric at each sample time point (fig. S2). In the current study, we used this large database of sequences associated with clinical phenotypes to search for known antigen-specific TCRβ sequences.

**Table 1.** Demographic and immunologic characteristics of study participants with TCRβ sequencing. NA, not applicable.

	Control	Case	New onset	Overall *P* value*
	*n* = 25 (100 samples)	*n* = 29 (114 samples)	*n* = 143 (143 samples)	
**Age,** mean (SD) in years				
Sample 1	1.9 (1.2)	2.1 (1.4)	NA	NA
Sample 2	5.2 (2.3)	5.5 (2.4)	NA	NA
Sample 3	6.6 (2.4)	6.7 (3.0)	NA	NA
Sample 4	11.7 (3.8)	12.2 (4.1)	12.1 (4.2)	0.89
T1D diagnosis	NA	12.8 (4.3)	12.1 (4.2)	NA
**Gender**				
Female % (number)	36% (*n* = 9)	38% (*n* = 11)	45% (*n* = 65)	0.56
**Race/ethnicity**				0.89
Non-Hispanic	84% (*n* = 21)	90% (*n* = 26)	85% (*n* = 122)	
Hispanic	16% (*n* = 4)	10% (*n* = 3)	14% (*n* = 20)	
Not reported	0% (*n* = 0)	0% (*n* = 0)	1% (*n* = 1)	
**Presence of islet autoantibodies**†, % (number)				
Sample 1	0% (*n* = 0)	7% (*n* = 2)	NA	NA
Sample 2	0% (*n* = 0)	62% (*n* = 18)	NA	NA
Sample 3	0% (*n* = 0)	93% (*n* = 27)	NA	NA
Sample 4	0% (*n* = 0)	86% (*n* = 25)	94% (*n* = 135)	<0.001
**HLA genotype,** % (number)				
DR4/X	28% (*n* = 7)	41% (*n* = 12)	44% (*n* = 63)	0.34
DR3/X	20% (*n* = 5)	17% (*n* = 5)	19% (*n* = 27)	1.0
DR3/4	36% (*n* = 9)	31% (*n* = 9)	27% (*n* = 39)	0.63
DRX/X	16% (*n* = 4)	10% (*n* = 3)	10% (*n* = 14)	0.61
**Productive TCRβ templates**‡				
Mean (SD) × 10^5^	1.49 (0.77)	1.42 (0.60)	1.58 (0.78)	0.54
Median × 10^5^	1.42	1.30	1.50	0.144
Range × 10^5^	0.16–4.42	0.13–4.19	0.10–3.81	

*P values for continuous variables use *t* test, categorical variables use Fisher’s exact test.

†At least one islet autoantibody includes those measured to insulin (IAA), glutamic acid decarboxylase (GAD), tyrosine phosphatase–related isletantigen-2 (IA-2), and zinc transporter 8 (ZnT8).

‡Values for templates are from those in all samples.

We curated a list of antigen-specific CD4 and CD8 TCRs reactive to either islet or viral proteins from the published literature (Fig. 1A). Inclusion criteria for manuscripts that identified islet antigen–specific TCR sequences included (i) indexed in PubMed and published between 2012 and 2022, (ii) identified full-length and paired TCRα chains (TRAV, TRAJ, and CDR3α) and TCRβ chains (TRBV, TRBJ, and CDR3β), and (iii) determined antigen specificity using one of the following methods: single-cell cloning, tetramer/multimer staining, x-ray crystallography, or generation of TCR transductants/avatars. For viral-specific TCRs, similar criteria were used, but there was a focus on those restricted to HLA class I and II molecules that are prevalent in T1D (e.g., HLA-A2, HLA-DR3/4, and HLA-DQ2/8). From 39 manuscripts, a total of 1743 antigen-specific TCRs were identified, with 1338 islet antigen–reactive and 405 viral-reactive sequences (data file S1). The islet antigen–specific CD4 TCRs included those responding to epitopes within PPI (*12*–*26*), GAD (*21*, *24*, *27*–*30*), hybrid insulin peptides (HIPs) (*31*–*34*), and other islet peptides (*35*, *36*) (Fig. 1C). The islet antigen specificities of CD8 TCRs included PPI (*17*, *22*, *24*, *37*, *38*), ZnT8 (*24*, *39*), and islet-specific glucose-6-phosphatase (IGRP) (*24*, *40*–*43*). Viral specificities were predominantly CD8 and included influenza, coxsackievirus B, Epstein-Barr virus (EBV), CMV, and adenovirus (ADV) (*28*, *37*, *42*–*50*). We searched for these antigen-specific TCRβ sequences (identical V, J, and CDR3) in our bulk TCRβ sequence database that included 357 peripheral blood samples from the three patient cohorts. Expectedly, most of the sequences (73.5%) were private and not shared in any of these samples; however, 26.5% of the antigen-specific sequences were public (i.e., present in at least one sample, *n* = 463 sequences; Fig. 1C), while thereof 14.9% were deemed frequent by being present in at least 1% of samples (≥4 samples, *n* = 259 sequences; Fig. 1C). Overall, our approach of searching for islet- and viral antigen–specific TCR sequences from bulk TCRβ repertoires in peripheral blood identified hundreds of shared and frequent antigen-specific TCRβ sequences in individuals at risk or with T1D.

### Viral antigen–specific CD8 TCRβ sequences are present across cohorts and ages

**Fig. 2.**
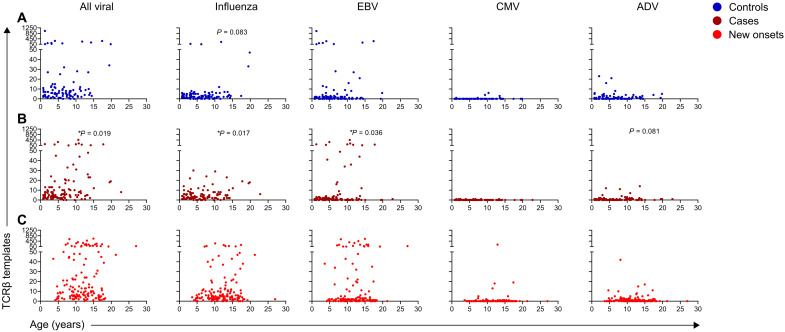
Viral antigen–specific CD8 TCRβs are present across ages and patient cohorts. Scatterplots displaying viral antigen TCRβ sequence total template numbers for each sample relative to age in years for (**A**) controls, (**B**) cases that progressed to clinical T1D, and (**C**) patients with new-onset T1D. Each dot represents the sum of all templates for TCRβ sequences with a given antigen specificity in one sample. Plots for all viral antigen–specific TCRβ sequences include both CD4 sequences (*n* = 2) and CD8 sequences (*n* = 103), while the remaining plots display viral CD8 TCRβ sequences grouped by antigen specificity; influenza (*n* = 51), EBV (*n* = 43), CMV (*n* = 2), and ADV (*n* = 7). *P* values were calculated using mixed-effects models to account for multiple measurements in cases and controls; linear regression was used for new-onset T1D. *P < 0.05.

Exposure to viral pathogens is common during childhood and adolescence, with resultant T cell specificities in peripheral circulation. As such, we tracked viral-specific TCRβ sequences across individuals focusing on the 27% (103 of 388) of sequences from CD8 TCRs that were shared and frequent; only two viral CD4 TCRβ sequences were found to be public and frequent in the cohort samples (Fig. 1C). Among all viral-specific sequences that were public and frequent (*n* = 105), TCRβ templates were present within each cohort and across ages (Fig. 2). All viral-specific templates increased with age in cases (*P* = 0.019; Fig. 2). In controls, there was a trend toward having more CD8 influenza-reactive TCRβ templates with age (*P* = 0.083; Fig. 2A), while CD8 influenza-reactive TCRβ template numbers was correlated to age in cases that progressed to T1D (*P* = 0.017; Fig. 2B). Both EBV- and ADV-specific TCRβ templates tended to increase with age in those progressing to T1D (*P* = 0.036 and 0.081, respectively; Fig. 2B). Although certain virus-specific TCRβ templates increased with age in cases, there was no correlation with risk of developing T1D. In the new-onset T1D cohort, viral-reactive TCRβ templates were also present, especially those directed against influenza and EBV (Fig. 2C). Collectively, these results indicate that viral-reactive TCRβ sequences can be readily detected in peripheral blood during childhood and adolescence.

### There are differential patterns of islet antigen–specific TCRβ sequences during progression to T1D

**Fig. 3.**
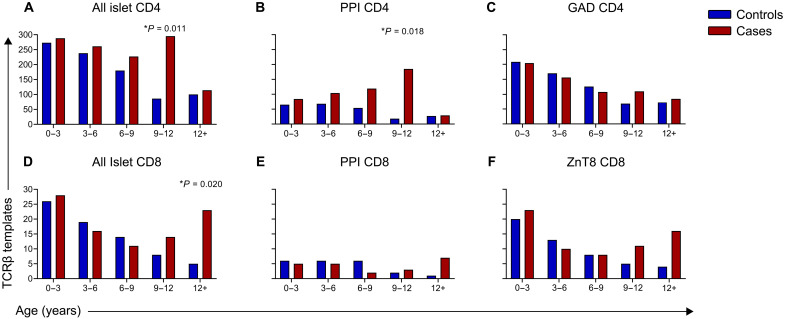
Differential patterns of islet antigen–specific TCRβ sequences during progression to T1D. Bar graphs showing the total number of TCRβ sequence templates in controls (blue) and preclinical T1D cases (red) within defined age groups. (**A**) Results for all islet antigen–reactive CD4, (**B**) PPI-reactive CD4, and (**C**) GAD-reactive CD4. The bottom panel of graphs depicts (**D**) all islet antigen–reactive CD8, (**E**) PPI-reactive CD8, and (**F**) ZnT8-reactive CD8 TCRβ sequence templates. Sample numbers for each age bin: 0 to 3 (controls, *n* = 24; cases, *n* = 26), 3 to 6 (controls, *n* = 27; cases, *n* = 30), 6 to 9 (controls, *n* = 24; cases, *n* = 26), 9 to 12 (controls, *n* = 14; cases, *n* = 17), and 12+ (controls, *n* = 11; cases, *n* = 15). *P* values were calculated using mixed-effects models to compare cases to controls within each age bin and to test for an interaction between group (cases and control) and time. None of the trajectories between cases and controls over time were statistically significant. *P < 0.05.

Next, we tracked the public and frequent islet antigen–specific CD4 TCRβ sequences, which encompassed 11% (136 of 1223) of all the searched islet antigen–reactive TCRs. The islet antigens recognized by the TCRs included insulin and its precursor, PPI, GAD, mixtures of islet peptides, and HIPs that are posttranslationally modified peptides implicated in T1D pathogenesis (*31*). In contrast to viral antigen–specific TCRβs, the presence of those specific for islet antigens had varying temporal dynamics, depending on the patient cohort and target antigen. Among all islet antigen–specific CD4 TCRβs, there were equivalent template numbers early in life in both controls and cases that progressed to clinical T1D. However, the number of templates steadily decreased over time in controls (Fig. 3, A to C, and fig. S3, A and B, for scatterplots by age), while those in cases were higher in number between the ages of 9 and 12 years (*P* = 0.011; Fig. 3A). This finding is primarily driven by CD4 TCRβs specific for PPI, as these decreased in template number in controls but accumulated in cases until 9 to 12 years of age (*P* = 0.018; Fig. 3B). The pattern for GAD-specific CD4 TCRβ sequences showed a decrease in controls with age, while there was more of a maintenance in template number of these sequences in cases over time (Fig. 3C and fig. S3, A and B).

Similar to the CD4 TCRβ sequences, public and frequent CD8 islet antigen–specific TCRβ chains comprised 16% (18 of 115) of the searched sequences. The antigens targeted by these CD8 TCRs included PPI, ZnT8, and IGRP. Cases and controls both had these TCRβ sequences early in life; however, template numbers tended to decline in controls and increase with age in cases (Fig. 3, D to F, and fig. S4, A and B). Differences in template numbers among all islet CD8 sequences between controls and cases occurred between the ages 9 and 12 years (*P* = 0.020; Fig. 3D). When analyzing temporal patterns by antigen, CD8 PPI TCRβs increased in cases over time, while decreasing in controls resulted in a trend toward statistical significance when comparing cases to controls over all ages (*P* = 0.10; Fig. 3E). The ZnT8-specific CD8 TCRβs showed decreased template numbers over time in controls and persistence in cases beyond 9 to 12 years of age (Fig. 3F). Together, these data indicate that CD4 and CD8 islet antigen–reactive TCRβ chain sequences were present with similar template numbers in T1D at-risk cases and controls very early in life with expansions in cases during the preclinical stages of disease and contractions in age/sex/HLA-matched controls throughout childhood.

We also examined islet antigen–specific CD4 and CD8 TCRβ chain sequences in a separate cohort of patients with new-onset T1D by age at clinical diagnosis. These TCRβs had higher template numbers in new-onset patients compared to the at-risk cases with preclinical T1D (fig. S5, A and D). Sequences for CD4 PPI TCRβs were present across all ages at diagnosis in childhood (fig. S3C), while CD8 PPI sequences were more predominant at older ages of diagnosis, >9 years of age (figs. S4C and S5, B and E). GAD CD4 TCRβ template numbers peaked at 6 to 9 years of age at T1D onset, while CD8 ZnT8 sequences had a bimodal distribution with greatest template numbers at 6 to 9 years old and then >12 years of age (fig. S5, C and F). These data indicate the presence of islet antigen–specific TCRβs in a separate cohort of patients with T1D.

### Identification of islet antigen–specific TCRβ chain sequences that are T1D disease–associated

**Fig. 4.**
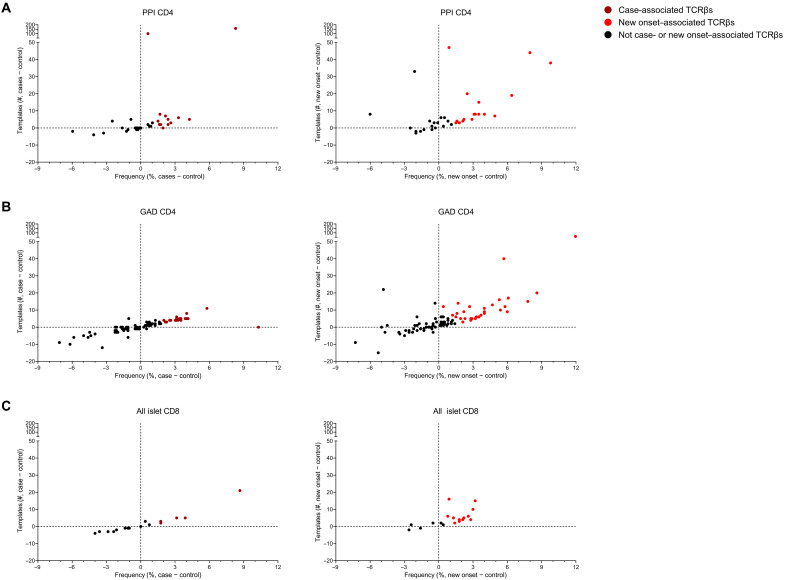
Identifying T1D disease–associated TCRβ chain sequences. Depicted are scatterplots with each TCRβ sequence plotted as the difference in sample frequency (%, *x* axis) versus the difference in templates (#, *y* axis) in preclinical T1D cases minus controls (left column) and in new-onset T1D minus controls (right column). Graphs are displayed by antigen specificity and T cell subset for (**A**) PPI CD4, (**B**) GAD CD4, and (**C**) all islet antigen–reactive CD8. Disease-associated TCRβs (*n* = 73 unique TCRβ sequences) are indicated in dark red (case-associated) or in light red (new onset–associated). Black circles represent those TCRβ sequences that are not case- or new onset–associated. Two data points are not displayed on the case plots to help visualize the data: [−12.23, 14.00 in (A)] and [−18.54, −34.00 in (B)].

Although islet antigen–reactive TCRβs were found in all three cohorts, we wanted to determine whether any TCRβ chain sequences were disease-associated, meaning that they were present at a substantially higher frequency (i.e., number of samples) and template number in preclinical disease (cases) or new-onset clinical T1D compared to controls. To compare the sample frequency and template number for each individual islet antigen–specific TCRβ sequence between cohorts, the values from controls were subtracted from those in case or new-onset T1D samples (Fig. 4). As depicted in Fig. 4, several PPI- and GAD-specific CD4 TCRβs (Fig. 4, A and B, respectively) were more frequent in case or new-onset samples compared to control samples. In addition, these TCRβ sequences had higher template numbers in cases and new onsets relative to controls. Those TCRβs with the largest differences in sample frequency and/or template number compared to controls were deemed disease-associated (Fig. 4, A and B, respectively). Furthermore, there were also islet antigen–specific CD8 TCRβs found to be disease-associated (Fig. 4C); however, there were fewer CD8 (*n* = 12) versus CD4 (*n* = 61) disease-associated TCRβs. Notably, several CD4 and CD8 islet antigen–specific TCRβ chains were similar in sample frequency and template number between the cohorts or even higher in controls relative to cases or new onsets (e.g., GAD-specific CD4 TCRβs in Fig. 4B); these TCRβ sequences were not deemed to be associated with preclinical or clinical T1D onset.

Next, we aimed to address the question: Is the expected HLA restriction of a disease-associated TCRβ sequence detected in a given sample matched to the donor HLA haplotype? As all of the participants across our three patient cohorts have HLA-DR and HLA-DQ typing (data file S2), we focused on matching the HLA-DR or HLA-DQ restriction to the disease-associated CD4 TCRβ sequences in table S1. HLA matching for each disease-associated CD4 TCRβ ranged from 48 to 100% (all identical matches; table S1). In summary, 79% (1174 of 1485) of the TCRβ sequences matched the donor HLA-DR-DQ type from which the disease-associated CD4 TCR was isolated and identified. These data indicate that there is a reasonable relationship between the TCRβ sequence and HLA class II expressed by the study participants in our cohorts. Together, these results indicate that there were 73 islet antigen TCRβ sequences deemed disease-associated because of their presence at higher sample frequencies and template numbers in the case cohort before disease onset and/or in new-onset T1D individuals at clinical diagnosis compared to age/sex/HLA-matched controls who did not develop islet autoantibodies or clinical disease (table S1).

### There are temporal changes within individual disease-associated TCRβ sequences during T1D development

**Fig. 5.**
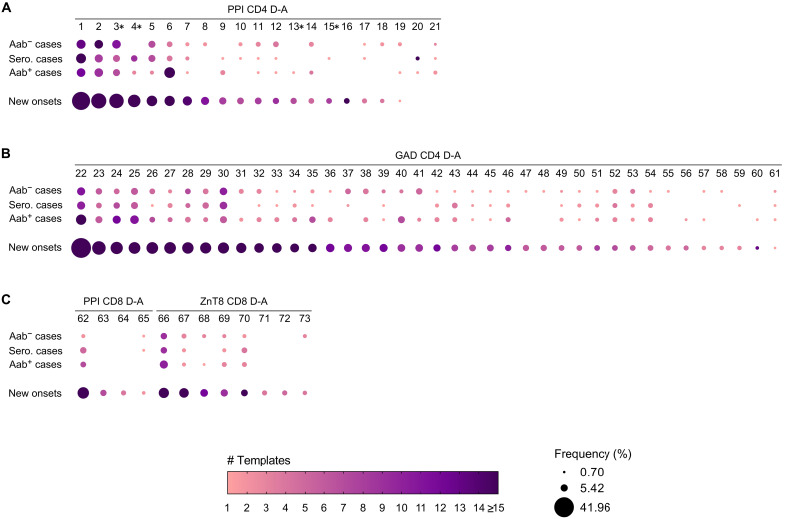
Temporal changes within individual disease-associated islet antigen TCRβ sequences during T1D development. Shown are multivariable plots for each disease-associated (D-A) TCRβ, grouped by antigen specificity and T cell subset for (**A**) PPI CD4 (*n* = 21 TCRβ sequences), (**B**) GAD CD4 (*n* = 40), and (**C**) PPI CD8 (*n* = 4), and ZnT8 CD8 (*n* = 8). Case samples are grouped in the first three rows by seroconversion status: before islet autoantibody seroconversion (Aab^−^), at islet autoantibody seroconversion, and after islet autoantibody positivity (Aab^+^). Results from the new-onset T1D cohort are shown in the bottom row. Dot size depicts the sample frequency within a patient cohort for each disease-associated TCRβ sequence, while a darker color depicts a higher number of TCRβ chain templates in case or new-onset T1D samples. *Disease-associated TCRβ sequence numbers 3, 4, 13, and 15 respond to HIPs.

Next, we examined when each of the 73 individual disease-associated TCRβ chains was present during the stages of T1D development: before islet autoantibody seroconversion, at seroconversion, after seroconversion, and at clinical diagnosis (Fig. 5). Twenty-one of the 73 disease-associated TCRβ chains were derived from CD4 PPI TCRs, and nearly all of these 21 TCRβs were present at higher sample frequencies and template numbers in the new-onset cohort compared to cases during preclinical T1D progression (TCRs 1 to 21; Fig. 5A). Most of these PPI TCRβs spanned all stages of T1D development. Four of the PPI TCRs are restricted to HIPs (#3, 4, 13, and 15 in Fig. 5 and table S1), which are formed within lysozymes of pancreatic β cells by the fusion of an insulin peptide fragment to another β cell peptide (*31*, *51*). T cell responses to these posttranslationally modified epitopes are implicated in T1D pathogenesis (*52*), and the HIP TCRβ sequences are present in both cases and new-onset T1D samples. There were 40 CD4 GAD-specific disease-associated TCRβs, with similar expression patterns to the CD4 PPI-specific sequences, in that new onsets expressed these TCRβs at much higher sample frequencies and template numbers than the cases during disease progression (TCRs 22 to 61; Fig. 5B). Disease-associated CD8 TCRβs were PPI or ZnT8 specific, and these were also expressed at varying stages of T1D development with higher frequencies and template numbers in the new-onset T1D cohort (TCRs 62 to 73; Fig. 5C).

Several disease-associated TCRβ sequences were only expressed in new-onset T1D samples: one CD4 PPI-specific (#16), two CD8 PPI-specific (#63 and #64), and two CD8 ZnT8-specific TCRβs (#71 and #72) were expressed only in that cohort and not in at-risk cases. Only two PPI TCRβs (#20 and #21) were present in cases during preclinical T1D stages and not within the new-onset cohort. In general, the disease-associated TCRβs were highly expressed in patients with new-onset T1D at clinical diagnosis. This result is not unexpected, as the majority of the initial curated list of TCRs was from studies that sequenced TCRs from individuals with newly diagnosed or established T1D, representing approximately two of three of the islet antigen TCR sequences (data file S1). Together, the disease-associated islet antigen–specific TCRβs did not segregate to particular stages of preclinical T1D but were rather expressed at varying times and across the stages of T1D development.

### Higher numbers of unique disease-associated TCRβ sequences correlates to an earlier age at T1D onset

**Fig. 6.**
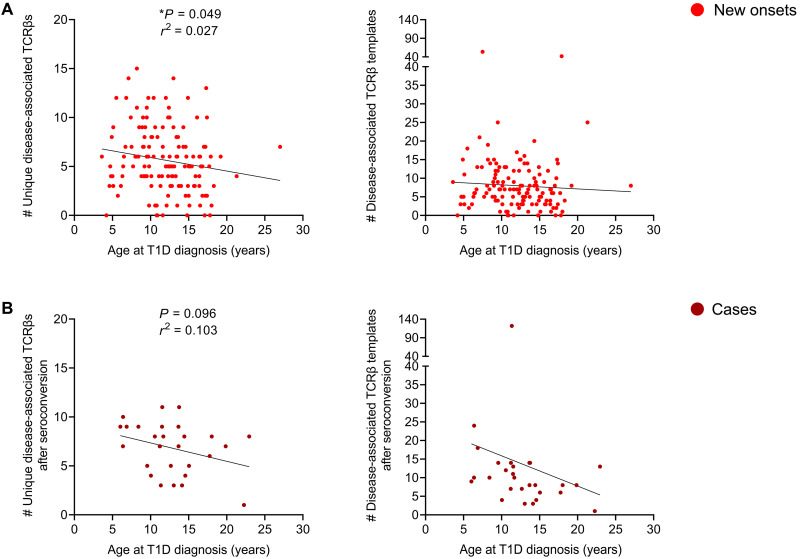
Total numbers of disease-associated islet antigen TCRβ sequences correlates to an earlier age at T1D diagnosis. (**A**) Scatterplots depicting the total number of unique disease-associated TCRβ sequences (left) or number of templates (right) versus age at T1D diagnosis for the new-onset cohort and (**B**) preclinical T1D cases for all samples after islet autoantibody seroconversion versus age at diagnosis. *P* values were calculated using linear regression. *P < 0.05.

We next wanted to determine whether the presence of disease-associated TCRβ sequences in a given individual was associated with age at clinical T1D onset. The ages of cases and patients with new-onset T1D at diagnosis ranged from childhood to early adulthood (3.6 to 27.0 years). In our new-onset T1D cohort, the presence of a higher total number of unique disease-associated TCRβs (i.e., number of the 73 disease-associated TCRβs) correlated to an earlier age of clinical T1D onset (*P* = 0.049; Fig. 6A). Similarly in cases, there was a trend toward correlation between the total number of disease-associated TCRβs expressed and age at clinical T1D onset (*P* = 0.096; Fig. 6B). Last, for both cohorts, the template number of disease-associated TCRβs tended to be higher with an earlier age at T1D diagnosis. These findings suggest that higher numbers of disease-associated islet antigen–specific TCRβ sequences in peripheral blood may correlate to a faster progression and younger age of clinical T1D onset.

## DISCUSSION

T cells play a vital role in disease pathogenesis for a variety of autoimmune diseases, including T1D. Our current study curated over 1700 antigen-specific TCRs from the literature and searched for the identical TCRβ chain sequences from bulk sequencing in peripheral blood DNA samples of children who progressed to T1D, age/sex/HLA-matched controls, and a cohort of patients with newly diagnosed T1D. Most of the TCR sequences were private and unique to a given individual, a phenomenon that has been demonstrated previously (*53*–*56*); however, we were able to identify shared and frequent TCRβ sequences from those known to recognize insulin or other β cell proteins and viral antigens. Expectedly, more viral-specific TCRβ chains were shared in the samples compared to islet antigen–specific TCRβ chains (27% versus 12%, respectively), and very large expansions of particular viral-specific TCRβs were seen in some individuals. As children are exposed to or infected with common viruses such as influenza, EBV, and CMV, T cell expansions occur in response. In contrast, relatively little is known about islet antigen–specific TCR repertoires during progression to clinical T1D.

Public islet antigen–specific TCRβ sequences varied in temporal dynamics between cases that progressed to T1D and controls, depending upon the targeted self-antigen. Both CD4 and CD8 PPI-responsive TCRβs accumulated throughout the preclinical period of T1D development, while reactivities to GAD and ZnT8 were present in similar numbers early in life but remained persistent in cases while declining in controls. Furthermore, we were able to identify 73 of these public islet antigen–specific TCRβs that were disease-associated, being present more frequently and with higher template numbers in at-risk cases and/or patients with new-onset T1D as compared to controls. The total number of unique T1D-associated TCRβ sequences correlated to age at clinical T1D onset, which complements the finding that the number of islet autoantibodies present in peripheral blood, predicts the eventual development of T1D (*57*). As the initial list of TCRs curated for this work was derived from multiple studies spanning numerous different patient samples separated in time and across geographical regions, the disease-associated TCRβ sequences identified here are likely to be prominent in many individuals developing T1D.

Most of the disease-associated TCRβ sequences were more frequent and present in higher template numbers in patients with newly diagnosed T1D with fewer numbers and some being absent during the preclinical period. One potential explanation for this observation is that expansion of islet antigen–specific T cells in new-onset individuals, compared to those with preclinical disease, may allow for these sequences to be more readily detected. The disease-associated TCRβ sequences should be monitored following clinical diagnosis to determine their presence and frequency in peripheral blood. A second explanation may stem from the composition of the patient cohorts from which the curated islet antigen–specific TCRs were obtained. Only a few studies have examined islet antigen–specific TCRs before disease onset (*11*, *21*, *58*), and only ~^1^/_3_ of the curated TCR sequences came from individuals with preclinical T1D. Hence, future studies are warranted to identify islet antigen–specific TCR sequences from individuals with preclinical T1D to better understand the role for these immunoreceptors in disease pathogenesis before clinical disease onset.

It is important to recognize several limitations to our approach. First, it should be noted that our study focused on matching identical TCRβ chain sequences from α/βTCRs with a known antigen specificity. However, both α and β chains contribute to the antigen specificity of a given T cell, and as TCR sequencing technologies continue to advance, it will be important to deep sequence paired α/βTCRs from a large number of T cells in the future. In addition, having paired α and β chains will allow for confirmation of TCR antigen specificity. However, sequencing just TCRβ chains can allow for the identification of viral-specific sequences directed against CMV and SARS-CoV-2 epitopes (*4*, *5*). Second, although several of the curated manuscripts provided phenotypic information about the T cells from which the antigen-specific TCRs were derived, the present study did not address functional characteristics of the cells harboring the TCRβ of interest. However, TCR sequence features within CDR3 have been shown to promote the development of self-reactive T cells (*59*–*61*). In addition, the presence of hydrophobic amino acid residues in the CDR3β influence T cell fate and are more frequent in regulatory TCRs, allowing for a scoring system to determine the TCR-intrinsic regulatory potential of CD4 TCRβ chain sequences (*62*). It is also becoming possible to link individual TCR sequences to functional genes at a single-cell level (*63*, *64*), which allows for the determination of the phenotypic characteristics of an islet antigen–specific TCR as T1D progresses and whether self-reactive TCRs found in controls are more likely to be regulatory T cells.

Therapies targeting T cells have been investigated for the ability to prevent T1D onset with teplizumab, an anti-CD3 monoclonal antibody, delaying the onset of clinical T1D by an average of 3 years compared to placebo (*65*, *66*). Additional T cell–modulating therapies and antigen-specific immunotherapies are being investigated clinically to preserve residual β cell function in T1D (*67*–*73*). The ability to monitor antigen-specific T cells involved in T1D pathogenesis directly from peripheral blood DNA before and after therapy holds promise as a potential biomarker of response for these treatments. In conclusion, our study provides evidence that islet antigen–specific and disease-relevant TCRs can be tracked in individuals progressing to clinical T1D, and these antigen receptors hold potential to aid in the timing and monitoring of immunotherapies for T1D prevention. Our results also provide a framework to monitor disease-relevant TCRs in a wide variety of autoimmune disorders before and after administration of therapeutics targeting antigen-specific T cells.

## MATERIALS AND METHODS

### Study cohorts

Three study cohorts with known clinical phenotypes and TCRβ immunoreceptor sequencing were used in this work (Fig. 1A), including (i) cases from the DAISY that progressed to clinical T1D, (ii) at-risk controls from DAISY that did not develop islet autoantibodies or clinical T1D, and (iii) a separate cohort of new-onset T1D patient samples. DAISY is an ongoing prospective birth cohort study that follows genetically at-risk children from birth for the development of islet autoantibodies and progression to clinical T1D (*74*). Cases and controls from DAISY had TCRβ sequencing performed using four peripheral blood samples collected during the study participation. All of the cases (*n* = 29, 114 samples) developed clinical T1D, and only one individual did not have islet autoantibodies but developed diabetes. Control samples (*n* = 25, 100 samples) were age- sex-, and HLA-matched to cases but did not develop islet autoantibodies nor develop clinical T1D (Table 1). Patients with new-onset T1D (*n* = 143, 143 samples) were recruited from the clinics at the Barbara Davis Center for Diabetes and were sampled on average 5 days (median 2 days) after beginning exogenous insulin treatment. Written informed consent was obtained from each participant or guardian when the participant was less than 18 years of age and the Colorado Multiple Institutional Review Board approved the study.

### Repository of TCRβ chain sequences

Deep sequencing of the TCRβ chain repertoires was performed using genomic DNA extracted from cryopreserved buffy coat samples as described previously (*11*). TCRβ chains were sequenced using the immunoSEQ Assay from Adaptive Biotechnologies. The identification of specific V and J genes was carried out according to the definitions established by the International ImMunoGeneTics collaboration (*75*). On average, approximately 150,000 productive TCRβ templates were obtained from each sample. The database containing all TCRβ sequencing results used in the current study is located at  https://clients.adaptivebiotech.com/pub/mitchell-2022-JCII and can be accessed using the freely available immuneACCESS feature from Adaptive Biotechnologies. The sequencing data with the associated clinical meta data are also freely available in the iReceptor Data Platform as part of the T1D TCR Repository (https://gateway.ireceptor.org/) (*76*).

### Curation of islet antigen–specific and viral TCR sequences

To curate a list of antigen-specific TCR sequences, we conducted a literature search in September 2022 using PubMed (https://pubmed.ncbi.nlm.nih.gov/). For islet antigen–specific CD4 and CD8 TCRs, inclusion criteria were the following: (i) Manuscripts published between 1 January 2012 and 30 September 2022 indexed in PubMed and searchable using broad keywords related to the current study, such as T1D and TCR; (ii) both α and β chain amino acid sequences TRAV, CDR3α, TRAJ, TRBV, CDR3β, and TRBJ for each TCR of interest were included in the publication; (iii) manuscripts included HLA restriction of the given TCR; (iv) the published work determined the antigen specificity of a given TCR by single-cell cloning, tetramer/multimer staining, x-ray crystallography, or the generation of a TCR transductant/avatar. The inclusion criteria for the viral antigen–specific TCRs were similar but focused on those restricted to HLA alleles that are frequent in T1D (e.g., HLA-A2, HLA-A1, HLA-DQ2/8, and HLA-DR3/4), with others restricted to HLA-B8, HLA-B7, or HLA-B35. A total of 39 manuscripts were used to curate a list of 1743 unique αβTCR sequences (data file S1).

### Matching known antigen-specific TCRβ sequences

Our curated list of antigen-specific TCR sequences included 1743 unique TCRβ sequences. The immunoSEQ Analyzer 3.0 platform from Adaptive Biotechnologies (http://adaptivebiotech.com/immunoseq) was used to search and match CDR3β sequences from the known antigen-specific TCR list to the bulk TCRβ sequences from the three study subject cohorts (*n* = 357 samples containing over 50 million sequences). Raw data from the CDR3β search were downloaded, and then an in-house bioinformatics pipeline was used to match TRBV and TRBJ for each CDR3β sequence at the amino acid level and determine the presence and template number for each identical V, J, and CDR3β sequence from a known antigen-specific TCRαβ in each sample. An antigen-specific sequence was determined to be shared if it was present in at least one sample from any cohort, and frequent antigen-specific TCRβ sequences had to be present in at least 1% of all samples (≥4 of 357 samples). Data file S2 provides the public antigen-specific TCRβ sequences at an individual sample level along with the clinical metadata including age, sex, ethnicity, islet autoantibodies present, and HLA type.

### Statistical analyses

Statistical analyses were performed using R software version 4.1 (R Core Team, Vienna) and GraphPad Prism 9.5 (GraphPad Software). *P* < 0.05 was considered significant. Continuous variables (age at time points) were compared using two-tailed *t* tests, and Fisher’s exact test was used for categorical variables (sex, race, ethnicity, presence of islet autoantibodies, and proportion of individuals with a given HLA type). Linear regression analyses were used to compare viral TCRβ templates to age and disease-associated TCR numbers or templates to age at T1D diagnosis. Mixed-effects models with an interaction between group and time were used to test whether the trajectories differed by group and to compare groups at each time point while accounting for the correlation of multiple measurements within a participant. One new-onset sample was not included in Figs. 2C and 6A, due to their age being a statistical outlier (Grubb’s outlier test).
