# Supplementary material for: Tracking DNA-based antigen-specific T cell receptors during progression to type 1 diabetes
Source: Sci Adv. 2023 Dec 8;9(49):eadj6975. doi: 10.1126/sciadv.adj6975 (PMC10708189; doi:10.1126/sciadv.adj6975)
Supplement: Supplementary file 1 — Figs. S1 to S5 Table S1 Legends for data files S1 and S2 [file sciadv.adj6975_sm.pdf]

Science Advances
AAAS

Supplementary Materials for

Tracking DNA-based antigen-specific T cell receptors during progression to type 1 diabetes

Angela M. Mitchell *et al.*

Corresponding author: Aaron W. Michels, aaron.michels@cuanschutz.edu

Sci. Adv. **9**, eadj6975 (2023)
DOI: 10.1126/sciadv.adj6975

The PDF file includes:

Figs. S1 to S5
Table S1
Legends for data files S1 and S2

Other Supplementary Material for this manuscript includes the following:

Data files S1 and S2

Supplementary Materials

Fig. S1: Similar numbers of productive TCRβ templates across samples and within each patient cohort.

Fig. S2: TCRβ clonality at sample time points.

Fig. S3: Islet antigen-specific CD4 TCRβ sequences across ages and patient cohorts.

Fig. S4: Islet antigen-specific CD8 TCRβ sequences across ages and patient cohorts.

Fig. S5: Islet-antigen reactive TCRβ sequences are present in new onset type 1 diabetes.

Table S1: Type 1 Diabetes disease-associated T cell receptors.

Data file S1: Curated list of viral and islet-antigen specific TCR sequences (excel file).

Data file S2: Public antigen-specific TCR sequences by sample with associated clinical metadata (excel file).

Fig. S1

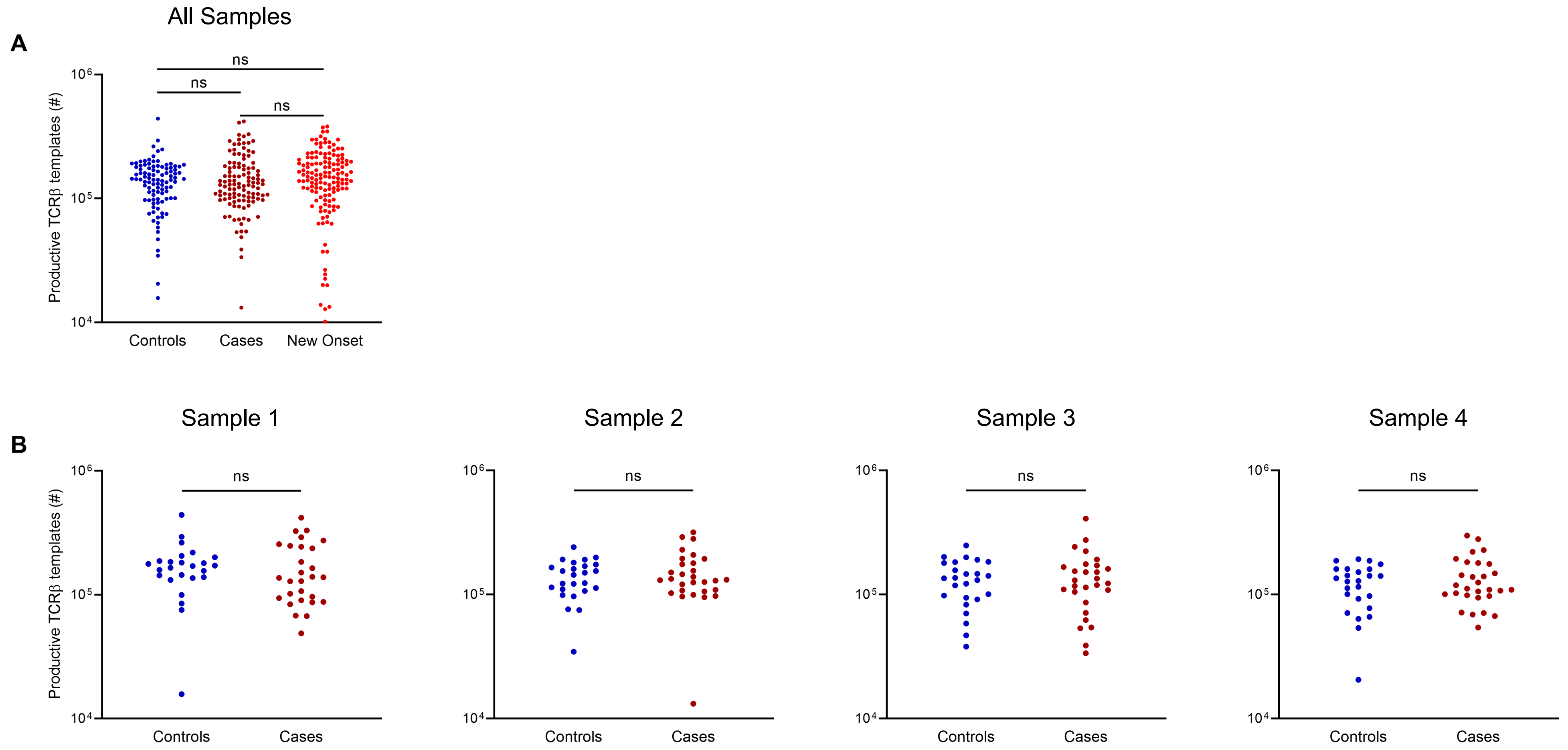

Supplemental Fig. S1: Similar numbers of productive TCRβ templates across samples and within each patient cohort. (**A**) Dot plots showing number of productive TCRβ templates for samples from controls (blue), cases (red), and new-onset T1D (light red) samples. (**B**) Dot plots displaying number of productive TCRβ templates per sample for controls and cases at each sample time point. P-values were calculated using mixed-effects models to account for multiple measurements.

Fig. S2

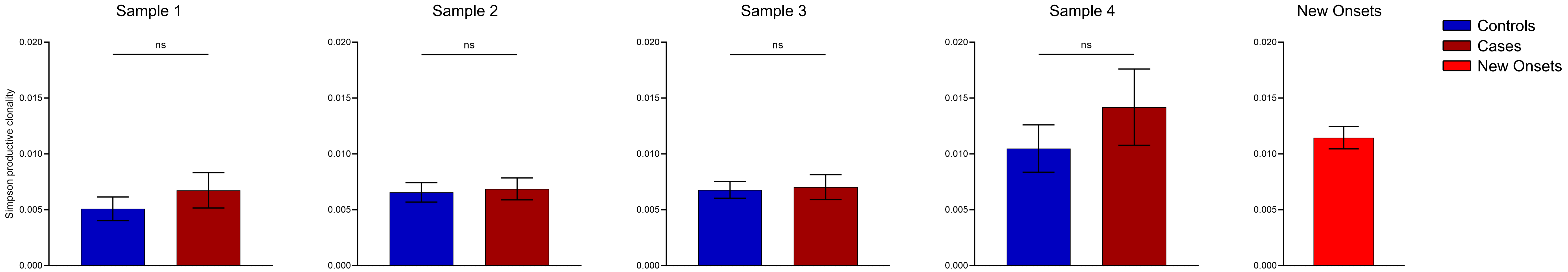

Supplemental Fig. S2: TCRβ clonality at samples time points. Plots showing Simpson productive clonality at each sample for controls (blue), cases (red), and new-onset T1D (light red). Controls, cases, and new onset T1D have similar ages at sample 4. P-values were calculated using Mann-Whitney tests comparing controls to cases at each sample.

Fig. S3

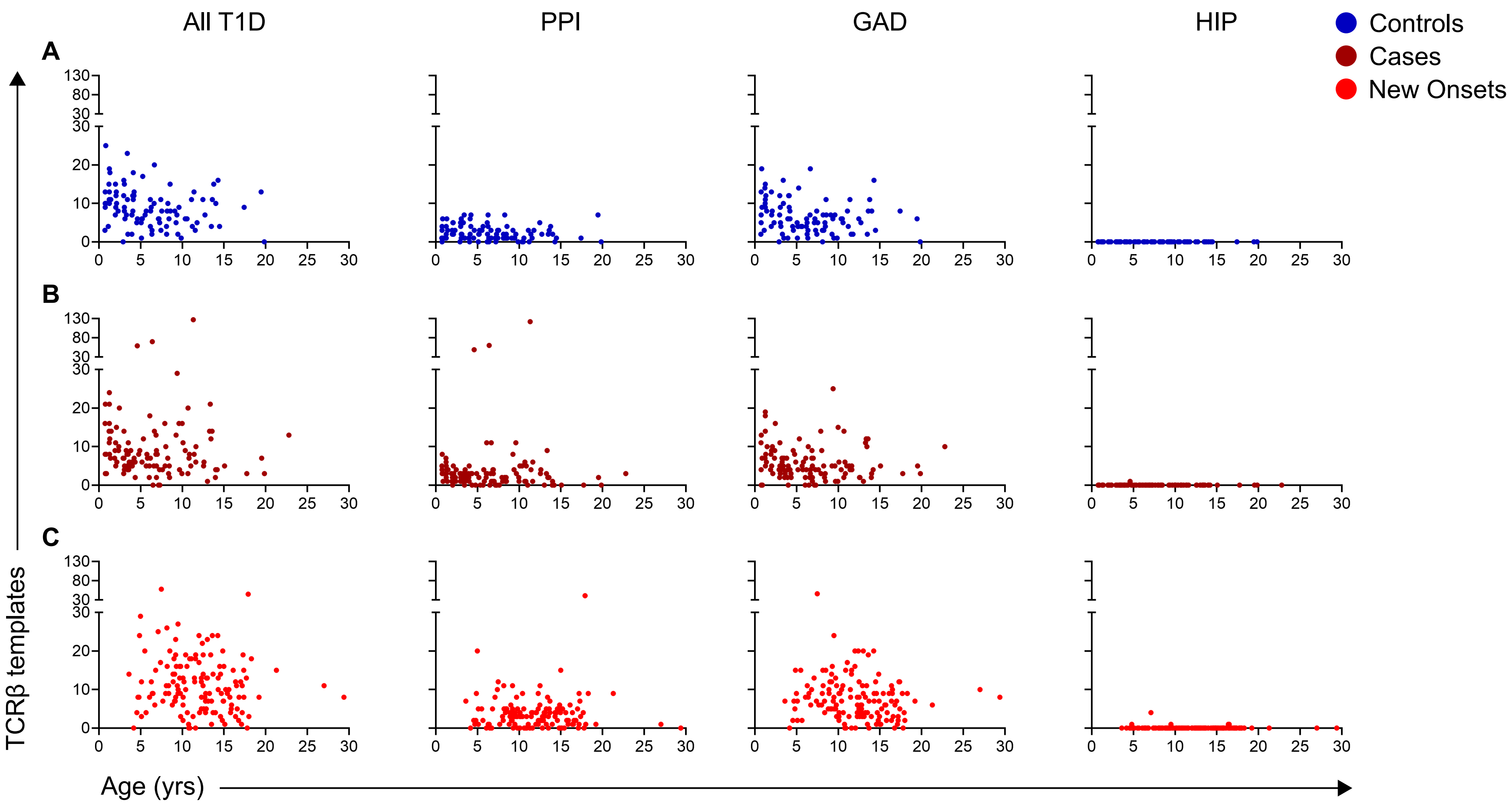

Supplemental Fig. S3: Islet antigen-specific CD4 TCRβ sequences across ages and patient cohorts. Scatterplots displaying public and frequent islet antigen-specific CD4 TCRβ sequence total template numbers for each sample relative to age in years for (**A**) controls, (**B**) cases, and (**C**) new-onset T1D patients. Each dot represents the sum of all templates for TCRβ sequences with a given antigen specificity in one sample. Plots for all T1D antigen-specific CD4 TCRβ include sequences across specificities (n=136), while the remaining plots display CD4 TCRβ sequences grouped by antigen specificity; PPI (n=31), GAD (n=100), and HIP (n=5).

Fig. S4

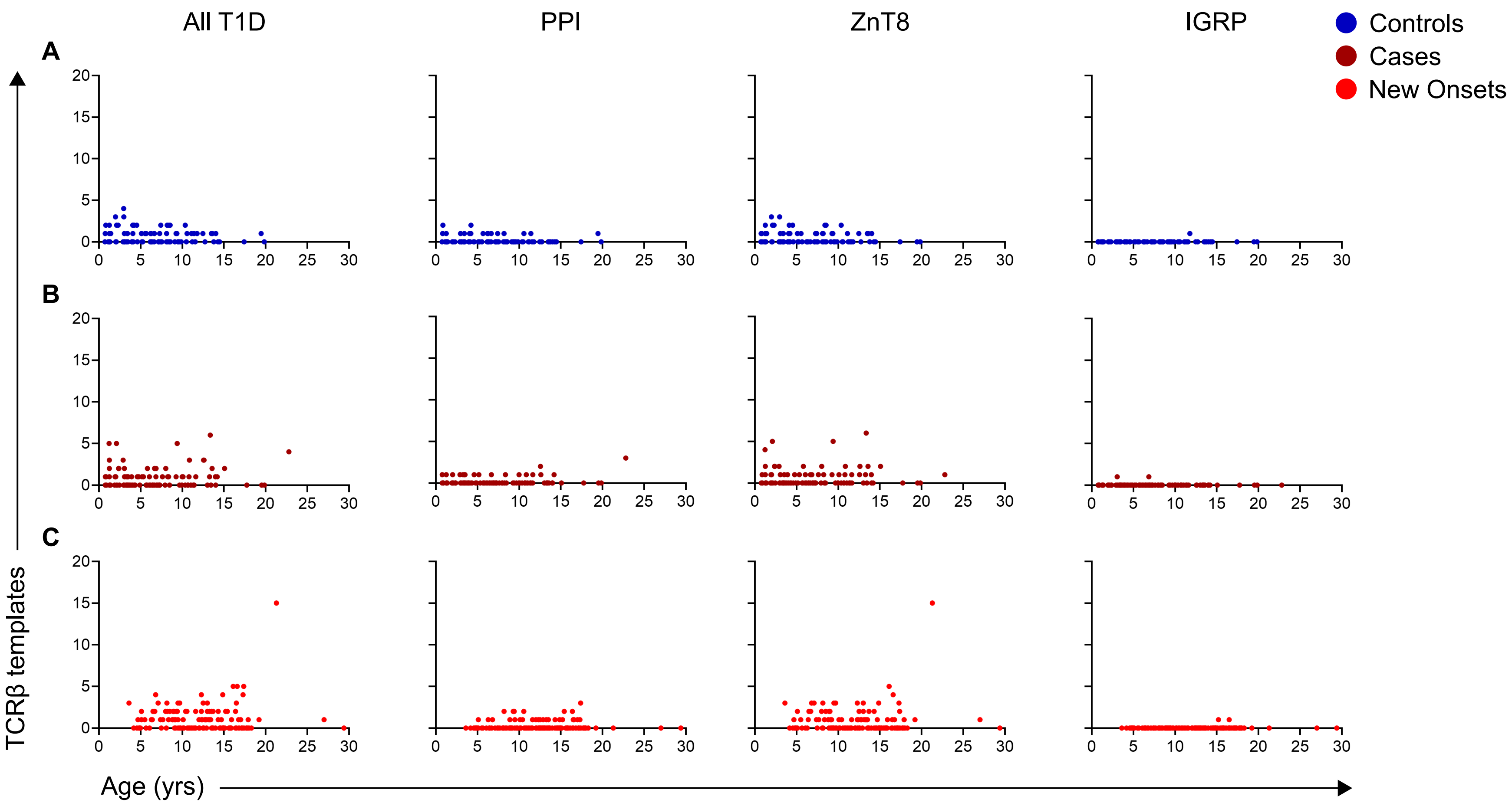

Supplemental Fig. S4: Islet antigen-specific CD8 TCRβ sequences across ages and patient cohorts. Scatterplots displaying islet antigen CD8 TCRβ sequence total template numbers for each sample relative to age in years for (**A**) controls, (**B**) cases, and (**C**) new-onset T1D patients. Each dot represents the sum of all templates for TCRβ sequences with a given antigen specificity in one sample. Plots for all T1D antigen-specific CD8 TCRβ include sequences across specificities (n=18), while the remaining plots display CD8 TCRβ sequences grouped by antigen specificity; PPI (n=6), ZnT8 (n=11), and IGRP (n=1).

Fig. S5

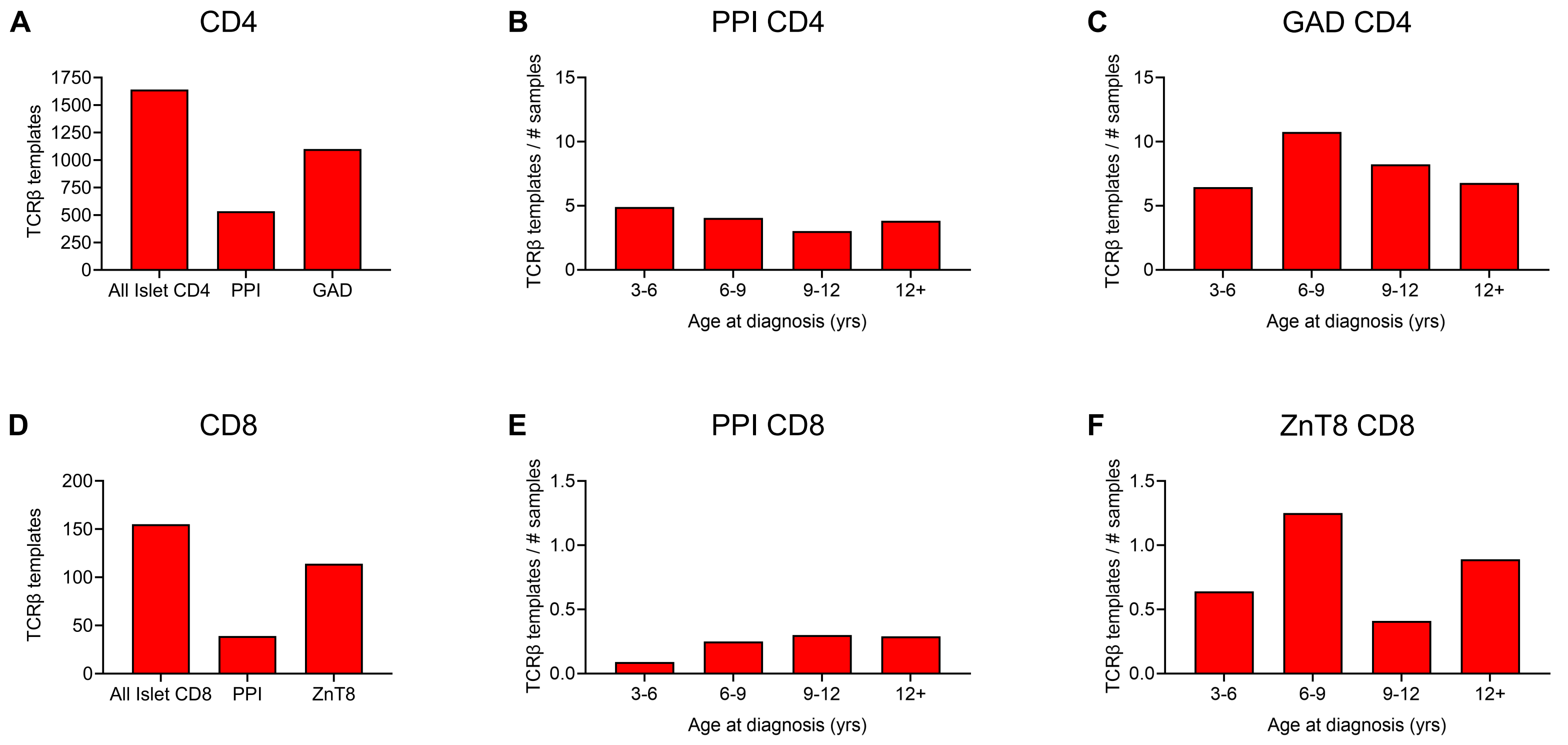

Supplemental Fig. S5: Islet antigen-reactive TCRβ sequences are present in new-onset type 1 diabetes. Displayed are bar graphs indicating the total number of TCRβ templates in new-onset T1D samples. (**A**) Total template numbers for all islet antigen CD4 TCRβ sequences. (**B**) PPI-reactive and (**C**) GAD-reactive CD4 TCRβ templates are displayed by age group and normalized to the number of samples in each age bin. (**D**) Total template numbers for all islet antigen CD8 TCRβ sequences. (**E**) PPI-reactive and (**F**) ZnT8-reactive CD8 TCRβ templates are displayed by age group and normalized to the number of samples in each age bin. Sample numbers for each age group: 3-6 (n = 11), 6-9 (n = 19), 9-12 (n = 37), 12+ (n = 76).

Table S1. Type 1 Diabetes disease-associated T cell receptors.

TCR	Subset	Peptide	HLA* Restriction	Location	TRAV	CDR3α	TRAJ	TRBV	CDR3β	TRBJ	Confirmation Method(s)	Controls Freq. (%) Temp. (#)	Cases Freq. (%) Temp. (#)	New Onsets Freq. (%) Temp. (#)	HLA-DR-DQ Match (%)	Reference(s)
1	CD4	Proinsulin	DR3-DQ2<br>DR4-DQ8	Blood	03-01	CAVRDKTPFQKLVF	8-1	04-01	CASSQGGTEAFF	1-1	Clone	27.00<br>42	28.95<br>42	34.97<br>86	88	Heninger et al. (21)
2	CD4	Proinsulin	DR3-DQ2<br>DR4-DQ8	Blood	29/DV5	CAARPYNTGNQFYF	49-1	03-01	CASSRTDTQYF	2-3	Clone	22.00<br>24	23.68<br>32	24.48<br>44	90	Heninger et al. (21)
3	CD4	C-peptide - IAPP2 HIP	DQ8	Blood/Islets	26-01<br>26-01	CIVSHNAGNMLTF<br>CIVRVVTGANNLFF	39-1<br>36-1	05-01	CASSLERETQYF	2-5	Clone, tetramer, transductant, crystal structure	16.00<br>18	19.30<br>24	22.38<br>37	69	Pathiraja et al. (19)<br>So et al. (23)<br>Tran et al. (33)
4	CD4	C-peptide - IAPP2 HIP	DQ8	Blood/Islets	20-02	CAVQAGGNNRLAF	7-1	05-01	CASSLERDGYTF	1-2	Clone, tetramer, transductant, crystal structure	7.00<br>7	6.14<br>12	16.78<br>45	76	Pathiraja et al. (19)<br>Wang et al. (26)<br>Tran et al. (33)
5	CD4	Proinsulin	DR3-DQ2<br>DR4-DQ8	Blood	08-06	CAVNGGSNYQLTF	53-1	03-01	CASSQGGYTEAFF	1-1	Clone	11.00<br>11	13.16<br>18	11.89<br>58	79	Heninger et al. (21)
6	CD4	Insulin B:9-23	DQ8	Blood/Islets	12-03	CAILSGGYNKLIF	4-1	02-01	CASSAETQYF	2-5	Clone, transductant	11.00<br>11	19.30<br>24	11.19<br>17	69	Yeh et al. (24)<br>Anderson et al. (17)
7	CD4	Proinsulin	DR3-DQ2<br>DR4-DQ8	Blood	17-01	CATDATGTYKYIF	40-1	29-01	CSVRGTGELFF	2-2	Clone	6.00<br>6	4.39<br>6	9.09<br>14	96	Heninger et al. (21)
8	CD4	Proinsulin	DR3-DQ2<br>DR4-DQ8	Blood	08-01	CAVKGSDGQKLLF	16-1	18-01	CASSPPGTETQYF	2-5	Clone	3.00<br>3	2.63<br>3	6.99<br>11	94	Heninger et al. (21)
9	CD4	Proinsulin	DR3-DQ2<br>DR4-DQ8	Blood	08-03	CALQTGANNLFF	36-1	29-01	CSVRTANTEAFF	1-1	Clone	4.00<br>4	3.51<br>4	5.59<br>8	88	Heninger et al. (21)
10	CD4	PPI: 50-58	DQ8	Islets	25-01	CAGGFSDGQKLLF	16-1	20-01	CSARTEAYEQYF	2-7	Clone	0.00<br>0	2.63<br>3	4.90<br>7	100	Pathiraja et al. (19)
11	CD4	Proinsulin	DR3-DQ2<br>DR4-DQ8	Blood	12-01	CVXNYLNDYKLSF	20-1	06-05	CASSYLGANTGELFF	2-2	Clone	2.00<br>3	4.39<br>5	4.90<br>8	86	Heninger et al. (21)
12	CD4	Proinsulin	DR3-DQ2<br>DR4-DQ8	Blood	14/DV4	CAMREGPTAGNMLTF	39-1	06-06	CASSYLGYEQYF	2-7	Clone	1.00<br>1	5.26<br>6	4.20<br>9	92	Heninger et al. (21)
13	CD4	C-peptide - IAPP2 HIP	DQ8	Blood/Islets	26-01	CIAIYNFNKFYF	21-1	05-01	CASSLEASSYNSPLHF	1-6	Clone, tetramer, transductant, crystal structure	2.00<br>2	0.88<br>1	4.20<br>7	67	Pathiraja et al. (19)<br>Tran et al. (33)
14	CD4	Proinsulin	DR3-DQ2<br>DR4-DQ8	Blood	08-01	CAVNAGGSARQLTF	22-1	04-01	CASSLTRGETQYF	2-5	Clone	2.00<br>2	4.39<br>7	3.50<br>5	92	Heninger et al. (21)
15	CD4	C-peptide - IAPP2 HIP	DQ8	Islets	26-01	CIVRVEIQGAQKLVF	54-1	05-01	CASSLGPGLRETQYF	2-5	Clone, tetramer, transductant, crystal structure	0.00<br>0	0.88<br>1	3.50<br>8	83	Tran et al. (33)
16	CD4	Proinsulin: C19-A3	DR4	Blood	16-01<br>39-01	CALSGRRSGGSNYKLTF<br>CAVDSGGTSYGKLTF	53-1<br>52-1	18-01	CASSPGHRQETQYF	1-1	Clone	0.00<br>0	0.00<br>0	3.50<br>15	80	Beringer et al. (13)
17	CD4	Proinsulin: C19-A3	DR4	Blood	04-01	CLVGDFGSARQLTF	22-1	27-01	CASSFWGYNEQFF	1-1	Clone	1.00<br>1	1.75<br>2	2.80<br>4	71	Beringer et al. (13)
18	CD4	Proinsulin	DR3-DQ2<br>DR4-DQ8	Blood	08-03	CALQTGANNLFF	36-1	10-03	CAIRRGTGELFF	2-2	Clone	0.00<br>0	1.75<br>2	2.10<br>10	100	Heninger et al. (21)
19	CD4	Proinsulin: C19-A3	DR4	Blood	05-01	CAESPNAGNNRKLIW	38-1	19-01	CASSIGPTQPQHF	1-1	Clone	1.00<br>1	2.63<br>3	0.70<br>1	80	Beringer et al. (13)
20	CD4	Insulin B:9-23	DQ8	Blood	21-01	CAVKRTGGSYIPTF	6-1	11-02	CASSSFWGSDTGELFF	2-2	Clone, transductant	2.00<br>3	2.63<br>103	0.00<br>0	80	Tan et al. (23)<br>Yeh et al. (24)
21	CD4	PPI: 87-101	DRB1*04:04	Islets	09-02	CALRTDRGSTLGRLYF	18-1	11-02	CASSLQSSYNSPLHF	1-6	Transductant	2.00<br>2	3.51<br>6	0.00<br>0	83	Landry et al. (18)
22	CD4	GAD65 (557I)	DR4	Blood	23/DV6	CAAIPLTGNEKLTF	48-1	14-01	CASSQGLNTEAF	1-1	Tetramer	30.00<br>37	28.95<br>42	41.96<br>97	70	Eugster et al. (27)
23	CD4	GAD65 (557I)	DR4	Blood	08-03	CAVGDQAGTALIF	15-1	05-01	CASSLAMNTEAFF	1-1	Tetramer	11.00<br>14	13.16<br>17	19.58<br>34	67	Eugster et al. (27)
24	CD4	GAD65 (557I)	DR4	Blood	36/DV7	CAVETYNNNDMRFF	43-1	18-01	CASSPTNTGELF	2-2	Tetramer	10.00<br>13	15.79<br>24	16.08<br>30	65	Eugster et al. (27)
25	CD4	GAD65 (557I)	DR4	Blood	12-01	CVVIKTGANNLFF	36-1	29-01	CSVEGGSSYEQYF	2-7	Tetramer	9.00<br>13	19.30<br>23	14.69<br>63	77	Eugster et al. (27)
26	CD4	GAD65 (557I)	DR4	Blood	04-01	CATASWVVSIPALGVNSF	44-1	04-01	CASSQGGGNQPQHF	1-5	Tetramer	13.00<br>16	11.40<br>16	14.69<br>30	66	Eugster et al. (27)
27	CD4	GAD65	DR3-DQ2<br>DR4-DQ8	Blood	24-01	CASSTGTASKLTF	44-1	28-01	CASSPTGYEQYF	2-7	Clone	10.00<br>11	7.89<br>11	14.69<br>24	80	Heninger et al. (21)
28	CD4	GAD65	DR3-DQ2<br>DR4-DQ8	Blood	41-01	CAVGANNNDMRF	43-1	28-01	CASSLGDQPQHF	1-5	Clone	10.00<br>16	9.65<br>16	13.99<br>27	83	Heninger et al. (21)
29	CD4	GAD65	DR3-DQ2<br>DR4-DQ8	Blood	08-03	CAVDNAGNMLTF	39-1	07-09	CASSPGGGYTF	1-2	Clone	8.00<br>9	10.53<br>13	13.29<br>25	85	Heninger et al. (21)
30	CD4	GAD65	DR3-DQ2<br>DR4-DQ8	Blood	02-01	CAVEDQNDMRF	43-1	24-01	CATSDQETQYF	2-5	Clone	15.00<br>20	18.42<br>25	13.29<br>22	95	Heninger et al. (21)
31	CD4	GAD65	DR3-DQ2<br>DR4-DQ8	Blood	26-02	CILRHDNAGHMFAF	39-1	04-01	CASSQVQETQYF	2-5	Clone	9.00<br>11	4.39<br>5	11.19<br>20	90	Heninger et al. (21)
32	CD4	GAD65	DR3-DQ2<br>DR4-DQ8	Blood	10-01<br>10-01	CVGGDRGSTLGGLYF<br>CVVRDRSSTLGRLYF	18-1<br>18-1	25-01	CASSERETQYF	2-5	Clone	2.00<br>2	6.14<br>7	9.79<br>17	87	Heninger et al. (21)
33	CD4	GAD65 (557I)	DR4	Blood	08-03	CAVGRGRLYNNKYF	21-1	14-01	CASSQTDTQYF	2-3	Tetramer	4.00<br>4	4.39<br>5	9.79<br>16	74	Eugster et al. (27)
34	CD4	GAD65 (557I)	DR4	Blood	03-01	CAVRDGAGKSTF	27-1	18-01	CASSPRQGGETQYF	2-5	Tetramer	3.00<br>5	5.26<br>8	8.39<br>15	48	Eugster et al. (27)
35	CD4	GAD65 (557I)	DR4	Blood	03-01	CAPTSTGFQKLVF	8-1	07-02	CASSFSSGNTIYF	1-3	Tetramer	5.00<br>6	7.02<br>10	7.69<br>18	63	Eugster et al. (27)
36	CD4	GAD65 (557I)	DR4	Blood	17-01	CATDGSSNGGKLIF	37-2	20-01	CSARDLRNEQFF	2-1	Tetramer	4.00<br>5	3.51<br>4	7.60<br>12	84	Eugster et al. (27)
37	CD4	GAD65 (557I)	DR4	Blood	08-02	CVVSPRGYNKLIF	4-1	28-01	CASSPPGPYEQYF	2-7	Tetramer	1.00<br>2	4.39<br>7	6.99<br>11	56	Eugster et al. (27)
38	CD4	GAD65	DR3-DQ2<br>DR4-DQ8	Blood	29/DV5	CDSGDMRF	43-1	05-04	CASSSGTVNTEAFF	1-1	Clone	3.00<br>3	6.14<br>7	6.99<br>11	90	Heninger et al. (21)
39	CD4	GAD65	DR3-DQ2<br>DR4-DQ8	Blood	16-01	CALSPLEYGNKLVF	47-1	02-01	CASSEQGNEQFF	2-1	Clone	3.00<br>3	3.51<br>6	6.99<br>12	100	Heninger et al. (21)
40	CD4	GAD65 (557I)	DR4	Blood	03-01	CAVRRSGGSYIPTF	6-1	25-01	CASSELYEQYF	2-7	Tetramer	3.00<br>3	7.02<br>11	6.29<br>9	85	Eugster et al. (27)
41	CD4	GAD65	DR3-DQ2<br>DR4-DQ8	Blood	08-02	CAVSEAGAGSYQLTF	28-1	06-05	CASSYSGTGYYEQYF	2-7	Clone	4.00<br>4	7.02<br>8	6.29<br>9	86	Heninger et al. (21)
42	CD4	GAD65	DR3-DQ2<br>DR4-DQ8	Blood	08-03	CAVGGSSNTGKLIF	37-2	03-01	CASSQDYTGELFF	2-2	Clone	4.00<br>4	5.26<br>6	5.59<br>12	89	Heninger et al. (21)
43	CD4	GAD65 (557I)	DR4	Blood	25-01	CAGSGGYQKVTF	13-2	05-01	CASSLVGRDTEAFF	1-1	Tetramer	3.00<br>3	7.02<br>8	4.90<br>8	61	Eugster et al. (27)
44	CD4	GAD65	DR3-DQ2<br>DR4-DQ8	Blood	01-01	CAVNSGGYQKVTF	13-2	12-03	CASRLQGNQPQHF	1-5	Clone	2.00<br>2	2.63<br>4	4.90<br>7	92	Heninger et al. (21)
45	CD4	GAD65	DR3-DQ2<br>DR4-DQ8	Blood	04-01	CLVDQTGANNLFF	36-1	05-01	CASSLGLANYGYTF	1-2	Clone	3.00<br>3	2.63<br>3	4.90<br>8	77	Heninger et al. (21)
46	CD4	GAD65	DR3-DQ2<br>DR4-DQ8	Blood	08-02	CAVSGYNTDKLIF	34-1	02-01	CASSEQGEQYF	2-7	Clone	3.00<br>3	6.14<br>9	4.20<br>10	81	Heninger et al. (21)

47	CD4	GAD65	DR3-DQ2<br>DR4-DQ8	Blood	24-01	CASHTGTASKLTF	44-1	28-01	CASSSLAYEQYF	2-7	Clone	1.00<br>1	0.88<br>1	4.20<br>6	88	Heninger et al. (21)
48	CD4	GAD65 (557I)	DR4	Blood	10-01	CVVGGSTLGGLYF	18-1	28-01	CASSYRGEQYF	2-7	Tetramer	1.00<br>1	0.88<br>1	4.20<br>6	50	Eugster et al. (27)
49	CD4	GAD65 (557I)	DR4	Blood	22-01	CVSGTASKLTF	44-1	05-04	CASSPGQGNTIYF	1-3	Tetramer	0.00<br>0	3.51<br>5	3.50<br>6	78	Eugster et al. (27)
50	CD4	GAD65 (557I)	DR4	Blood	29/DV5	CAADASYSGGGADGLTF	45-1	03-01	CASSLQGTYEQYF	2-7	Tetramer	0.00<br>0	3.51<br>4	3.50<br>6	89	Eugster et al. (27)
51	CD4	GAD65	DR3-DQ2<br>DR4-DQ8	Blood	08-04	CAVSASNTNAGKSTF	27-1	18-01	CASSQGDYGYTF	1-2	Clone	2.00<br>2	5.26<br>6	3.50<br>8	85	Heninger et al. (21)
52	CD4	GAD65 (557I)	DR4	Blood	23/DV6	CAASMEGSARQLTF	22-1	18-01	CASSRGTEAFF	1-1	Tetramer	4.00<br>5	7.89<br>10	3.50<br>7	72	Eugster et al. (27)
53	CD4	GAD65	DR3-DQ2<br>DR4-DQ8	Blood	17-01	CAGNDYKLSF	20-1	18-01	CASSPGLGYNEQFF	2-1	Clone	3.00<br>3	6.14<br>8	3.50<br>6	93	Heninger et al. (21)
54	CD4	GAD65 (557I)	DR4	Blood	02-01	CAVGALIKAAGNKLTF	17-1	20-01	CSAPRGTYEQYF	2-7	Tetramer	4.00<br>4	7.02<br>8	3.50<br>5	59	Eugster et al. (27)
55	CD4	GAD65 (557I)	DR4	Blood	08-04	CVLGKAAGNKLTF	17-1	20-01	CSATTGTVNTEAFF	1-1	Tetramer	0.00<br>0	0.88<br>1	2.80<br>4	80	Eugster et al. (27)
56	CD4	GAD65 (557I)	DR4	Blood	27-01	CAGMNYGGSQGNLIF	42-1	19-01	CASSTVVNTEAFF	1-1	Tetramer	0.00<br>0	0.88<br>2	2.80<br>5	100	Eugster et al. (27)
57	CD4	GAD65 (557I)	DR4	Blood	23/DV6	CAPRGSQGNLIF	42-1	27-01	CASSLLRGTGELFF	2-2	Tetramer	0.00<br>0	1.75<br>2	2.10<br>3	80	Eugster et al. (27)
58	CD4	GAD65	DR3-DQ2<br>DR4-DQ8	Blood	02-01	CAVNHGSSNTGKLIF	37-2	28-01	CASSLRGRGNTIYF	1-3	Clone	0.00<br>0	0.88<br>1	2.10<br>3	100	Heninger et al. (21)
59	CD4	GAD65 (557I)	DR4	Blood	10-01	CVGGERGSTLGGVFF	18-1	25-01	CASSEEGNQPQHF	1-5	Tetramer	0.00<br>0	0.88<br>1	2.10<br>3	75	Eugster et al. (27)
60	CD4	GAD65 (557I)	DR4	Blood	39-01	CAVINTNAGKSTF	27-1	13-01	CASSSLSSYEQYF	2-7	Tetramer	1.00<br>1	0.88<br>1	1.40<br>13	100	Eugster et al. (27)
61	CD4	GAD65	DR3-DQ2<br>DR4-DQ8	Blood	26-01	CIVRVNPGGSEKLVF	57-1	29-01	CSARTNYGYTF	1-2	Clone	0.00<br>0	2.63<br>4	0.70<br>1	100	Heninger et al. (21)
62	CD8	PPI: 1-11	C*03:04	Islets	12-03	CAMSALGNFGNEKLTF	48-1	19-01	CASSIAGGNEQFF	2-1	Transductant	11.00<br>12	11.40<br>15	13.99<br>22	NA	Anderson et al. (17)
63	CD8	PPI: 31-41,<br>PPI: 34-41	A*02:01	Islets	26-02	CILTDNYGQNFVF	26-1	27-01	CASSLIGLNTEAFF	1-1	Transductant	2.00<br>2	0.88<br>1	4.20<br>7	NA	Anderson et al. (17)
64	CD8	PPI: 1-11	C*03:04	Islets	08-04	CAVSDQGSGYSTLTF	11-1	28-01	CASSWTANQPQHF	1-5	Transductant	0.00<br>0	0.00<br>0	2.80<br>4	NA	Anderson et al. (17)
65	CD8	PPI: 3–11	A*24:02	Blood	05-01	CAEPSGNTGKLIF	23-1	07-09	CASSLHHEQYF	2-7	Tetramer	0.00<br>0	1.75<br>2	1.40<br>2	NA	Kronenberg et al. (37)
66	CD8	ZnT8: 186-194	A*02:01	Blood	16-01	CAPTGANNL	36-1	28-01	CASSFGNEQFF	2-1	Multimer, clone	8.00<br>8	16.67<br>29	11.19<br>23	NA	Culina et al. (39)
67	CD8	ZnT8: 186-194	A*02:01	Blood		Not resolved		19-01	CASSAGQGAYEQYF	2-7	Multimer, clone	9.00<br>10	6.14<br>7	9.79<br>16	NA	Culina et al. (39)
68	CD8	ZnT8: 186-194	A*02:01	Blood	01-02	CASKDSNYQLIW	33-1	19-01	CASSIGQGSQPQHF	1-5	Multimer, clone	5.00<br>7	2.63<br>4	6.29<br>12	NA	Culina et al. (39)
69	CD8	ZnT8: 186-194	A*02:01	Blood	29-01	CAAIGNRDDKIIF	30-1	27-01	CASSFGNSYEQYF	2-7	Multimer, clone	3.00<br>3	6.14<br>8	5.59<br>9	NA	Culina et al. (39)
70	CD8	ZnT8: 186-194	A*02:01	Blood	38/DV1	CALRSGYALNF	41-1	25-01	CASSDQETQYF	2-5	Multimer, clone	4.00<br>4	7.89<br>9	4.90<br>20	NA	Culina et al. (39)
71	CD8	ZnT8: 186-194	A*02:01	Blood	35-02	CAGTRNNLFF	36-1	19-01	CASGGSSYEQYF	2-7	Multimer, clone	1.00<br>1	0.00<br>0	2.80<br>4	NA	Culina et al. (39)
72	CD8	ZnT8: 186-194	A*02:01	Blood	29/DV5	CAASGTLTTSGTYKYIF	40-1	04-02	CASSQEGTAYEQYF	2-7	Multimer, clone	1.00<br>1	0.00<br>0	2.80<br>5	NA	Culina et al. (39)
73	CD8	ZnT8: 186-194	A*02:01	Blood	12-03	CAMSAEGNYQLIW	33-1	06-05	CASSIGSGNTIYF	1-3	Multimer, clone	0.00<br>0	1.75<br>3	2.10<br>4	NA	Culina et al. (39)

PPI = preproinsulin, IAPP = islet amyloid polypeptide, HIP = hybrid insulin peptide, GAD65 = 65-kD isoform of glutamic acid decarboxylase, ZnT8 = zinc transporter 8, NA = not applicable
Frequency is the number of samples in a patient cohort that has the disease-associated TCRβ sequence.
*DQ8 consists of the alleles *DQA1*03:01-DQB1*03:02* and *DQA1*03:03-DQB1*03:02*
*DQ2 consists of the subtypes: *DQA1*05:01-DQB1*02:01*, *DQA1*02:01-DQB1*02:02, DQA1*03:01-DQB1*02:02*, or *DQA1*03:02-DQB1*02:02*
*DR3 indicates *DRB1*03:01*
*DR4 indicates *DRB1*04:01*, unless otherwise specified.
** HLA-DR-DQ matching is the frequency of samples that have a given TCRβ sequence that match the reference HLA restriction.
